# The triggers of situational syncope do not influence the head-up tilt test response and prognosis

**DOI:** 10.1093/europace/euae208

**Published:** 2024-08-06

**Authors:** Vincenzo Russo, Erika Parente, Angelo Comune, Anna Rago, Gerardo Nigro, Michele Brignole

**Affiliations:** Cardiology and Syncope Unit, Department of Translational Medical Sciences, University of Campania ‘Luigi Vanvitelli’—Monaldi Hospital, 80131, Naples, Italy; Cardiology and Syncope Unit, Department of Translational Medical Sciences, University of Campania ‘Luigi Vanvitelli’—Monaldi Hospital, 80131, Naples, Italy; Cardiology and Syncope Unit, Department of Translational Medical Sciences, University of Campania ‘Luigi Vanvitelli’—Monaldi Hospital, 80131, Naples, Italy; Cardiology and Syncope Unit, Department of Translational Medical Sciences, University of Campania ‘Luigi Vanvitelli’—Monaldi Hospital, 80131, Naples, Italy; Cardiology and Syncope Unit, Department of Translational Medical Sciences, University of Campania ‘Luigi Vanvitelli’—Monaldi Hospital, 80131, Naples, Italy; IRCCS Istituto Auxologico Italiano, Faint & Fall Research Centre, Department of Cardiology, S. Luca Hospital, 20149, Milan, Italy

**Keywords:** Syncope, Situational syncope, Head-up tilt test, Cardioinhibitory syncope, Recurrence syncope

## Abstract

**Aims:**

The study evaluated the positivity rate, haemodynamic responses, and prognosis in terms of syncopal recurrence among patients with situational syncope (SS) stratified according to the underlying situational triggers.

**Methods and results:**

We retrospectively evaluated all consecutive patients with SS who underwent nitroglycerine (NTG)-potentiated head-up tilt test (HUTT) at Syncope Unit of the University of Campania ‘Luigi Vanvitelli’—Monaldi Hospital from 1 March 2017 to 1 May 2023. All patients were followed for at least one year. The study population was divided according to the underlying triggers (micturition, swallow, defaecation, cough/sneeze, post-exercise). Two hundred thirty-six SS patients (mean age 50 ± 19.3 years; male 63.1%) were enrolled; among them, the situational trigger was micturition in 109 patients (46.2%); swallow in 32 (13.6%) patients; defaecation in 35 (14.8%) patients; post-exercise in 41 (17.4%) patients; and cough/sneeze in 17 (7.2%) patients. There were no significant differences in baseline clinical characteristics and HUTT responses between different situational triggers. The Kaplan–Meier analysis did not show a statistically different rate of syncope recurrence across patients stratified by baseline situational triggers (log-rank *P* = 0.21).

**Conclusion:**

Situational syncope appears to be a homogenous syndrome, and different triggers do not impact the HUTT response or syncope recurrence at 1 year.

## Introduction

Situational syncope (SS) is a form of reflex syncope following specific triggers/circumstances (micturition, swallow/defaecation, cough/sneeze, post-exercise, laughing, brass instrument playing).^1^ The head-up tilt test (HUTT) has high diagnostic accuracy, ∼71.1%, among patients with SS; moreover, it is useful for detecting hypotensive susceptibility or cardioinhibitory component of syncope. The type of response and positivity rate of HUTT among SS patients is similar to that observed in those with vasovagal syncope.^2^ The HUTT responses of the different SS triggers are poorly investigated, and no data are yet available about the syncope recurrence stratified according to the underlying situational triggers.^3^

## Methods

We aimed to evaluate the HUTT positivity rate, haemodynamic responses, and prognosis among 236 consecutive patients with SS who underwent nitroglycerine (NTG)-potentiated HUTT at Syncope Unit of the University of Campania ‘Luigi Vanvitelli’—Monaldi Hospital of Naples, from 1 March 2017 to 1 May 2023. All patients were followed for at least one year. According to our clinical practice, we performed HUTT in all patients with suspected reflex syncope, including those with SS, in order to confirm the diagnosis and to evaluate the main determinant of the transient loss of consciousness. The HUTT was performed according to the ‘Italian Protocol’ or ‘Fast Italian Protocol’.^4^ During the whole duration of the HUTT, all patients underwent continuous electrocardiographic monitoring and non-invasive beat-to-beat arterial blood pressure measurement (Task Force® monitor; CNSystem, Graz, Austria). The HUTT responses were classified according to the new VASIS classification.^5^ The normal distribution of the data was assessed using the Kolmogorov–Smirnov and Shapiro–Wilk tests. Continuous normally distributed variables were expressed as mean ± standard deviation (SD) and were compared by using permutation one-way analysis of variance test. Categorical variables were reported as numbers and percentages; and were compared with the Fisher–Freeman–Halton exact test.

The study population was dichotomized according to the different situational triggers (micturition, swallow, defaecation, cough/sneeze, post-exercise). The first syncopal episode after the HUTT evaluation was recorded and classified as SS or vasovagal syncope (VVS). This latter includes syncopal episodes due to orthostatic (standing) or emotional (fear, somatic/visceral pain, instrumentation, blood phobia) triggers. The syncope recurrence-free rates among the study groups during follow-up were evaluated with the Kaplan–Meier method and compared with the log-rank test. For all test, a *P* value of <0.05 was considered statistically significant.

### Ethics

This study was conducted according to the Declaration of Helsinki and approved by the institutional ethics committee of the University of Campania Luigi Vanvitelli—Monaldi Hospital (ID-168/02032021). Written informed consent for data collection was obtained from the patients at the time of execution of head-up tilt test.

## Results

Two hundred thirty-six patients with situational syncope (mean age 50 ± 19.3 years; male 63.1%) were included in the present study; among them, the situational trigger was micturition in 109 patients (46.6%); swallow in 32 (13.7%) patients; defaecation in 35 (15%) patients; post-exercise in 41 (17.5%) patients; and cough/sneeze in 17 (7.3%) patients. All patients had a single reproducible trigger for their SS. There were no significant differences in baseline clinical characteristics and HUTT responses between different situational triggers (*Table 1*). The time between the last spontaneous syncope and the HUTT was 54 ± 15 days. All SS patients received education, lifestyle modifications, and reassurance regarding the benign nature of their condition. In 55 patients (23.3%), the hypotensive therapy was reduced or discontinued; 15 patients (6.4%) received permanent pacemaker therapy; 11 patients (47.7%) received implantable loop recorder.

**Table 1:** Clinical characteristics and HUTT responses by situational triggers

	Total *n* = 236	Micturition *n* = 109	Swallow *n* = 32	Defaecation *n* = 35	Cough, sneeze *n* = 17	Post-exercise *n* = 41	*P*
Age (years), mean ± SD	50 ± 19.3	49.3 ± 19.4	51 ± 19.4	52 ± 19	53.9 ± 15.7	46.5 ± 18.4	0.72
Male sex, *n* (%)	149 (63.1)	69 (63.3)	19 (59.4)	18 (51.4)	12 (70.6)	29 (70.7)	0.45
Smoking, *n* (%)	88 (37.3)	41 (37.6)	15 (46.9)	14 (40)	7 (41.2)	11 (26.8)	0.07
Hypertension, *n* (%)	75 (31.8)	31 (28.4)	14 (43.7)	8 (22.9)	5 (29.4)	16 (39)	0.22
Diabetes mellitus, *n* (%)	20 (8.5)	10 (9.2)	3 (9.4)	1 (2.9)	0 (0)	6 (14.6)	0.33
Dyslipidaemias, *n* (%)	50 (21.2)	20 (18.3)	7 (21.9)	9 (25.7)	3 (17.6)	10 (24.4)	0.83
CAD, *n* (%)	7 (3)	4 (3.7)	1 (3.1)	1 (2.9)	1 (5.9)	0 (0)	0.68
CKD, *n* (%)	3 (1.3)	3 (2.7)	0 (0)	0 (0)	0 (0)	0 (0)	0.88
Number of syncopes during life, mean ± SD	1.5 ± 0.5	1.5 ± 0.5	1.5 ± 0.4	1.3 ± 0.5	1.4 ± 0.5	1.6 ± 0.5	0.36
Syncope without prodromes, *n* (%)	15 (6.4)	7 (6.4)	3 (9.4)	3 (8.6)	0 (0)	1 (2.4)	0.60
Traumatic syncope, *n* (%)	45 (19.1)	25 (22.9)	7 (21.9)	6 (17.1)	1 (5.9)	6 (14.6)	0.49
SBP, mmHg, mean ± SD	128.7 ± 21	127.3 ± 20	125.6 ± 20.2	121.5 ± 22.2	123.8 ± 15.7	127 ± 22.6	0.29
DBP, mmHg, mean ± SD	79.2 ± 13.7	79.7 ± 14.1	78.2 ± 13.9	76.7 ± 13.5	72.6 ± 9.2	79.1 ± 13.3	0.14
HR, bpm, mean ± SD	71.5 ± 13.7	68.9 ± 12	75 ± 13.7	73.9 ± 14.2	69.2 ± 16.1	75.5 ± 11.4	0.06
Alpha-blockers, *n* (%)	13 (5.5)	4 (3.7)	1 (3.1)	3 (8.6)	4 (23.5)	1 (2.4)	0.35
Beta-blockers, *n* (%)	20 (8.5)	8 (7.3)	3 (9.4)	1 (2.9)	2 (11.8)	6 (14.6)	0.38
CCA, *n* (%)	8 (3.4)	2 (1.8)	1 (3.1)	0 (0)	3 (17.6)	2 (4.9)	0.14
ACE-i/ARBs, *n* (%)	49 (20.8)	22 (20.2)	10 (31.1)	5 (14.3)	4 (23.5)	8 (19.5)	0.54
Diuretics, *n* (%)	18 (7.6)	9 (8.3)	4 (12.5)	1 (2.9)	1 (5.9)	3 (7.3)	0.66
HUTT positivity, *n* (%)	165 (69.9)	82 (75.2)	18 (56.2)	24 (68.6)	13 (76.5)	28 (68.3)	0.93
Mixed response, *n* (%)	113 (47.9)	57 (52.3)	11 (34.4)	17 (48.6)	10 (58.8)	18 (43.9)	0.82
CI response, *n* (%)	29 (12.3)	16 (14.7)	5 (15.6)	2 (5.7)	0 (0)	6 (14.6)	0.30
VD response, *n* (%)	22 (9.3)	9 (8.3)	2 (6.25)	4 (11.4)	3 (17.6)	4 (9.7)	0.6

ACE-I, angiotensin converting enzyme inhibitors; ARB, angiotensin II receptor blockers; CAD, coronary artery disease; CCA, calcium channel antagonist; CI, cardioinhibitory; CKD, chronic kidney disease; DBP, diastolic blood pressure; GI, gastrointestinal; HR, heart rate; HUTT, head up tilt test; SBP, systolic blood pressure; VD, vasodepressive.

During a mean follow-up of 336.9 ± 74.7 days, 42 patients (17.8%) experienced a recurrent syncopal episode due to situational (*n*: 15, 6.4%) or vasovagal (*n*: 27; 11.4%) trigger. Among patients with recurrent situational syncope, the trigger was micturition in nine patients (60%), swallow in two patients (13.3%), defaecation in one patient (6.7%), post-exercise in two patients (13.3%), and cough/sneeze in one patient (6.7%). Among patients with recurrent vasovagal syncope, the trigger was emotional in 22 patients (81.5%) and orthostatic in 5 patients (18.5%). In three patients, the trigger of recurrent syncope was different from the initial trigger.

**Figure 1:**
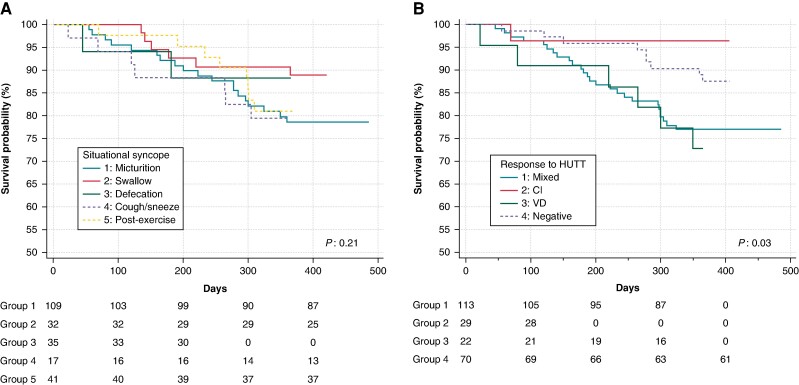
Kaplan–Meier curves for recurrent syncope free survival stratified by baseline situational triggers (*A*) and HUTT response (*B*).

Syncope recurred in 20.2% of patients with micturition syncope, 21% with swallow, 14.3% with defaecation, 23.5% with cough/sneeze, and 9.8% with post-exercise syncope at baseline evaluation. The Kaplan–Meier analysis did not show a statistically different rate of syncope recurrence across patients stratified by baseline situational triggers (log-rank *P* = 0.21) (*Figure 1A*).

The syncope recurrence rate based on the HUTT response was 23% (26/113) in patients with mixed syncope, 3.4% (1/29) in cardioinhibitory (CI) syncope, 27.3% (6/22) in vasodepressor (VD) syncope, and 12.7% (9/71) in those with negative HUTT. The Kaplan–Meier analysis showed a statistically different rate of syncope recurrence across patients stratified according to the HUTT-induced response (log-rank *P* = 0.03) (*Figure 1B*).

## Discussion

The main findings of the current study can be summarized as follows: SS patients show similar baseline clinical characteristics and HUTT responses, in terms of positivity rate and haemodynamic pattern, irrespective of the involved triggers. Among those patients with a recurrence of syncope during the subsequent follow-up, a majority had vasovagal syncope and, in those with a recurrence of SS, the situational trigger was sometimes different. No significant difference in the rate of syncope recurrence was shown across patients with different SS triggers at one-year follow-up. The risk of syncope recurrence was higher among SS patients with mixed and vasodepressor HUTT responses. These results are novel and expand our previous findings that did not show differences in HUTT positivity rate and type of responses between patients with situational and VVS.^2^ Based on these findings, we suggest that, despite the triggers and the afferent pathways involved in the various types of neurally mediated syncope are greatly different, all forms of VVS or SS have a similar response to HUTT. The orthostatic stress caused by HUTT can induce a similar positive response, even when the spontaneous triggers are quite different. We can infer that, in patients with neurally mediated syncope, the integration at the level of the central nervous system and its effect on the final efferent pathways are similar irrespective of the triggers and the afferent limb. In brief, the disturbance is inside the brain in any clinical form of reflex syncope.

## Limitations

Our results should be interpreted considering the limitations related to the study’s retrospective, observational, and single-centre nature; however, it is the only study evaluating the HUTT positivity rate, the haemodynamic responses, and the one-year follow-up of SS patients stratified according to the different triggers. We did not perform a prolonged monitoring of the study population, and there is no documentation about what happened during the spontaneous syncope; however, the diagnostic pathway is generally concluded in presence of a positive HUTT response. The low syncopal burden among our patients may be explained by the inclusion of patients with situational syncope alone, quickly referred to our Syncope Unit.

## Conclusions

Situational syncope appears to be a homogenous syndrome, and different triggers do not impact HUTT response or syncope recurrence at 1 year.

## Authors’ contributions

Conceptualization: V.R. and M.B.; methodology: V.R. and E.P.; software: E.P., A.C., and A.R.; validation: M.B. and G.N.; formal analysis: E.P., A.C., and V.R.; investigation: V.R. and A.R.; resources: G.N.; clinical investigation: A.C. and A.R.; data curation: V.R., E.P., and A.C.; writing original draft preparation: V.R. and E.R.; writing review and editing: V.R. and M.B.; visualization: M.B.; supervision: M.B. and G.N.; project administration: G.N.; guarantor: V.R.; all authors have read and agreed to the published version of the manuscript.

## Patient and public involvement

Patients and/or the public were not involved in the design, or conduct, or reporting, or dissemination plans of this research.

## Data Availability

Data are available on reasonable request. The data that support the findings of this study are available from the corresponding author up on reasonable request.
